# Monthly variations in growth pattern and condition factor of *Periophthalmodon septemradiatus* (Gobiiformes: Periophthalminae) living along the Bassac River in Viet Nam

**DOI:** 10.7717/peerj.13880

**Published:** 2022-08-12

**Authors:** Quang Minh Dinh, Ton Huu Duc Nguyen, Ngon Trong Truong, Diep Xuan Doan, Tien Thi Kieu Nguyen

**Affiliations:** 1 Department of Biology, School of Education, Can Tho University, Can Tho, Vietnam; 2 Department of Molecular Biotechnology, Biotechnology Research and Development Institute, Can Tho University, Can Tho, Vietnam; 3 Medicinal Chemistry, Hi-tech Agriculture & Bioactive Compounds Research Group, School of Engineering and Technology, Van Lang University, Ho Chi Minh City, Vietnam; 4 Department of Biology, An Khanh High School, Can Tho, Can Tho, Vietnam

**Keywords:** Fish growth, Isometry, Length-weight relationship, Mekong Delta, Negative allometry, Regression parameters

## Abstract

*Periophthalmodon septemradiatus* (Hamilton, 1822) is a unique mudskipper living in the mudbanks from estuarine to riverine regions in the Vietnamese Mekong Delta (VMD), but there is no data on its growth patterns and condition factors that are helpful for fish resource assessment and adaptation understanding. This study was conducted at five sites, from the lower (Soc Trang province) to middle (Can Tho city) and upper (An Giang province) reaches of Bassac River in VMD, to provide knowledge on growth patterns and condition factors to this mudskippers. Fish samples were caught using traps and hands for 24 months, from July 2017 to June 2019, at these five sites. The total length and weight of 3,417 individuals (1,340 females and 2,077 males) varied by sex, season and site (*p* < 0.001 for all cases). This species exhibited a positive allometric type as the slope (*b* = 3.06 ± 0.01) of the length-weight relationship (LWR) was >3 (*p* < 0.001) for both sexes. The growth pattern changed with sex as mudskipper showed isometry in females but positive allometry in males. Maturity also affected fish growth type since it shifted from negative allometry in immature groups to positive allometry in mature groups. The growth pattern of fish changed from isometry in the dry season to positive allometry in the wet season. The mudskipper showed isometry at the lower reaches in Soc Trang but positive allometry at the middle and upper reaches in Can Tho and An Giang. The condition factor (CF) did not change according to sex, size and season, but with month and site variables. The present environmental condition in these studied sites tended to be not good enough for this fish as CF (0.95 ± 0.01) was less than 1 (*p* < 0.001). The findings provided basic information on the growth and adaptation of *P. septemradiatus* being helpful in fish adaptation understanding and resource conservation in VMD.

## Introduction

The assessment and management of the fish population are related to the fish length-weight relationship (LWR) (Gonzalez Acosta, De La Cruz Agüero & De La Cruz Agüero, 2004; Mahmood et al., 2012; Jin et al., 2015). The regression parameters as the intercept (initial growth coefficient) and the slope (growth coefficient) of the LWR are helpful to determine the growth pattern of fish (Froese, 2006). The growth pattern can change with sexes, fish sizes, seasons and sites (Froese, Palomares & Pauly, 2000; Dinh et al., 2022a). Condition factor (CF) is an essential factor in the study of fish adaptation to the environment (Abdoli et al., 2009; Dinh, 2016). In addition, the CF is critical for confirming if fish health varies between fish species and places (Abdoli et al., 2009) and changes with fish sizes and seasons (Froese, 2006). The CF can increase or decrease during the reproductive cycle of fish depending on the spawning stage (Gomiero & Braga, 2005; Lam & Dinh, 2021b; Phan et al., 2021b). Besides, this sudden change in value also indicates a difference in the feeding habits of some fish species (Gomiero & Braga, 2003; Phan et al., 2021a).

Oxudercidae is one of the unique fish families, with most species breathing in terrestrial environments (Zhang et al., 2003). Many species in this family live in coastal or riverside mudflats (Jaafar, Lim & Chou, 2006; Lam & Dinh, 2021a). Oxudercidae comprises 42 species belonging to 10 genera globally, *e.g*., *Boleophthalmus boddarti*, *Scartelaos histophorus, Periophthalmodon septemradiatus, P. schlosseri, Periophthalmus chrysospilos* (Fricke, Eschmeyer & Fong, 2022). In the Mekong Delta of Viet Nam (VMD), *P. septemradiatus* is a unique mudskipper as it distributes from coastal saltwater to freshwater deep in major rivers (Diep, Dinh & Tran, 2014; Mai et al., 2019; Tran et al., 2020; Tran et al., 2021a). Besides Vietnam, this species is also found in India, Myanmar, Thailand, Malaysia, Papua New Guinea, and Indonesia (Kottelat et al., 1993; Khaironizam & Norma-Rashid, 2003; Froese & Pauly, 2022). The unique skin structure with a robust capillary system allows them to breathe the air when living out of the water (Zhang et al., 2003). It feeds mainly on detritus, *Dolichoderus* sp., *Uca* spp., molluscs, *Acetes* spp., and small fishes (Dinh et al., 2020b). This mudskipper spawns monthly a year-round in burrows made by males (Dinh et al., 2020a) and reaches 12.6 cm in total length and a maximum lifespan of ~6 years (Tran & Dinh, 2020). Males are more colourful than females, and male morphology can change from golden brown to iridescent black-green during spawning (Dinh, Tran & Nguyen, 2018). As some males have blue-violet spots on the snout and gills, *P. septemradiatus* is considered a potential species for aquarium pets (Dinh et al., 2020a). But there is no data on its growth pattern and condition factor in VMD. This research, therefore, was performed to provide an understanding of the growth patterns and CF of this mudskipper. The factors including sex, size, season and site affected the fish growth pattern and CF were also presented in the present study. The studied results will be helpful for fish resource assessment and adaptation knowing.

## Materials and Methods

### Fish collection and analysis

**Figure 1:**
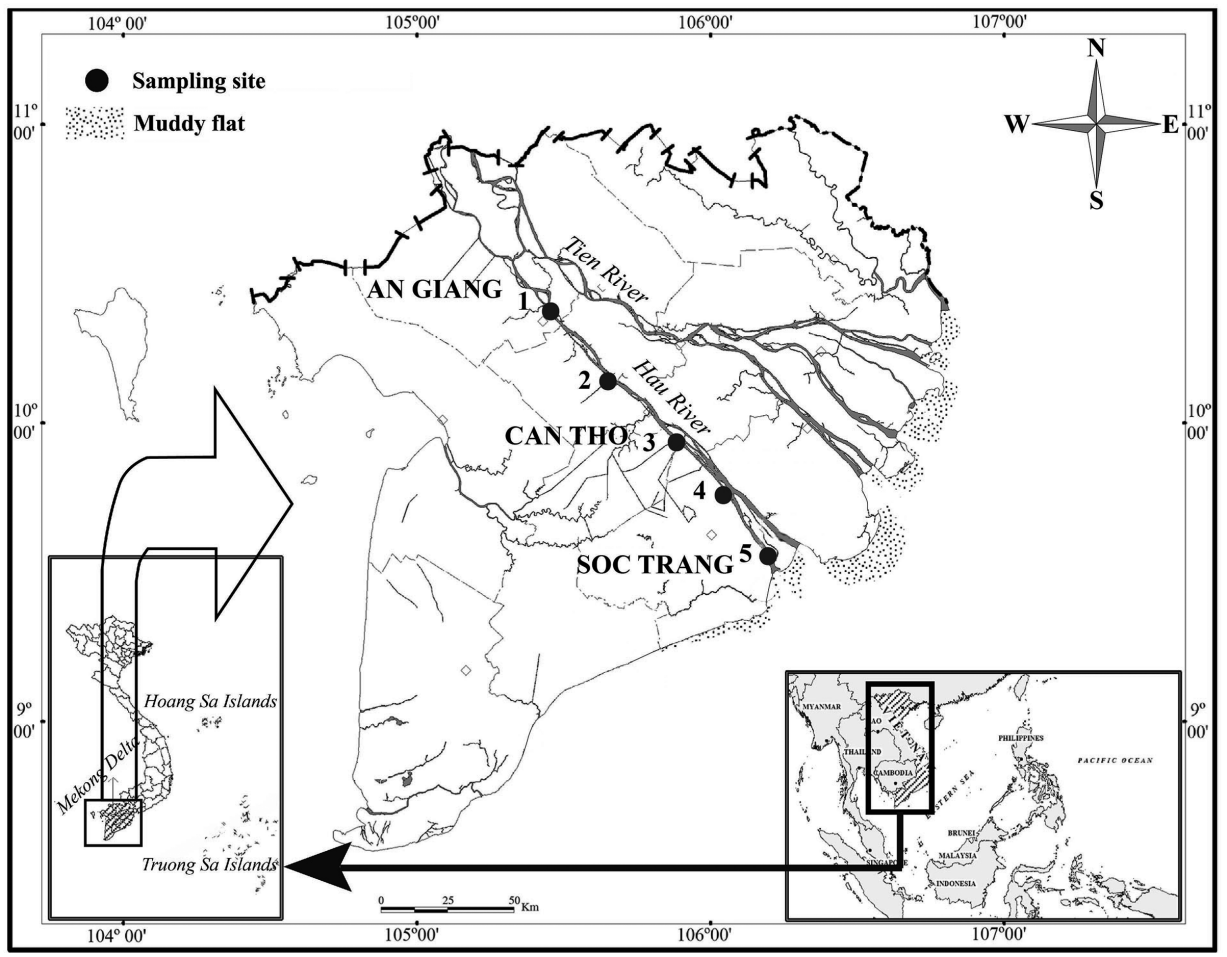
The sampling map in the Mekong Delta. (1: Long Xuyen, An Giang, 2: Thot Not, Can Tho, 3: Cai Rang, Can Tho, 4: Ke Sach, Soc Trang, and 5: Long Phu, Soc Trang) (Dinh, 2018).

*P. septemradiatus* were collected from five sites along the Bassac River in VMD, starting from Long Xuyen–An Giang (LXAG; 10°24′03.54″N 105°25′10.82″E) to Thot Not – Can Tho (TNCT; 10°12′07.17″N 105°34′43.89″E), Cai Rang–Can Tho (CRCT; 9°59′45.06″N 105°48′22.73″E), Ke Sach – Soc Trang (KSST; 9°49′52.4″N 105°59′44.5″E), and Long Phu, Soc Trang (LPST; 9°42′55.4″N 106°04′28.4″E) (Fig. 1). Fish samples were caught monthly using traps and hands from July 2017 to June 2019 (the dry season is from January to May with rare rain and the wet season is from June to December with heavy rain). After fishing, specimens were anaesthetized with MS222 before preserving in 5% formalin solution and transporting to the laboratory for analysis. The use of fish in the present research was approved by The Council for Science and Education, School of Education, Can Tho University under the Animal Welfare Assessment number: BQ2017-01/KSP.

The total length (TL) and weight (W) were measured and weighted using a ruler and an electronic balance to the nearest 0.1 cm and 0.01 g, respectively. Fish was sexed using their external traits, *e.g*., the dorsal fin of males is longer, larger, and more colourful than females, and the genital papilla is a triangle in males and oval in females (Dinh et al., 2020a). The formula W = *a* × TL^*b*^ was used to determine the relationship between TL and W (*a* and *b*: the intercept (initial growth coefficient) and the slope (growth coefficient), respectively) (Ricker, 1973). Fish showed isometric growth when *b* = 3; allometric growth when *b* ≠ 3 (Froese, 2006). The CF was determined by Le Cren (1951) function: *CF* = 100 × *W*/*TL*^*b*^ (*b* is the slop value taken from the LWR).

### Data analysis

The variation of TL, W, *b* and CF by sex, fish size and season was determined by a t-test. One-way ANOVA with Tukey *Post Hoc* was used to test the variation of TL, W, *b* and CF according to the sampling site. The fish size was divided into two groups using the length at first maturity (L_m_), documented by Dinh et al. (2020a). Accordingly, if fish with TL < L_m_ (L_m_ from LPST to LXAG was 8.2, 8.1, 7.5, 8.1, 9.2 cm TL for males and 6.6, 6.8, 6.1, 6.8 and 7.2 cm TL for females, respectively), was marked as an immature group and the revised case for mature group. The interaction of sex × fish size, sex × season, sex × sampling site, fish size × season, fish size × sampling site, season × sampling site affects the changes of *b* was determined by two-way ANOVA. The Pauly’s t-test was used to check whether *b* and CF were equal to 3 and 1, respectively (Ricker, 1975; Zar, 1999). The SPSS v21 was used for statistical processing, and all tests were set at a meaningful α = 5%. The value was presented in mean ± SE (standard error of the mean). According to Benjamini & Hochberg (1995) and McDonald (2014), Benjamini-Hochberg procedure was performed to lessen the Type I error of all tests.

## Results

### Length and weight distribution

The total length (TL) and weight (W) of 3,417 individuals (1,340 females and 2,077 males) collected at five sites from LPST to LXAG were 5.0–12.1 cm TL and 1.13–17.14 g W, respectively (Table 1). The TL varied by sex (*n* = 3,417, *df* = 3,415, *t* = −16.16, *p* < 0.001, *CI*_*95*%_ = (−0.89) to (−0.7)), and season (*n* = 3,417, *df* = 3,415, *t* = −4.99, *p* < 0.001, *CI*_*95*%_ = (−0.44) to (−0.19)). The TL of males (8.8 ± 0.1 cm) was significantly higher than that of females (8.0 ± 0.1 cm). Besides, season also affected the TL as this value in the dry season (8.6 ± 0.01 cm) were higher than in the wet season (8.3 ± 0.1 cm). The male W (6.41 ± 0.07 g) was significantly higher than in females (4.82 ± 0.07 g) (*n* = 3,417, *df* = 3,415, *t* = −15.47, *p* < 0.001, *CI*_*95%*_ = (−1.78) to (−1.38)). The W in the wet season (5.32 ± 0.12 g, *n* = 646) was lower than in the dry season (5.89 ± 0.06 g, *n* = 2,771) (*n* = 3417, *df* = 3415, *t* = −4.38, *p* < 0.001, *CI*_*95%*_ = (−0.83) to (−0.32)).

**Table 1:** The total length (TL, cm) and weight (W, g) of *Peirophthalmodon septemradiatus* caught from five sampling sites.

Months	Long Xuyen – An Giang				Thot Not – Can Tho				Cai Rang – Can Tho				Ke Sach – Soc Trang				Long Phu – Soc Trang			
	Female	Male	TL range	W range	Female	Male	TL range	W range	Female	Male	TL range	W range	Female	Male	TL range	W range	Female	Male	TL range	W range
July-17	5	6	5.6–11.6	1.39–13.04	13	4	7.4–11.1	3.32–11.66	7	15	5.95–9.9	1.40–7.26	10	20	6.4–10.7	1.77–11.38	6	30	5.8–11.6	2.01–12.86
August-17	8	7	6.6–11.5	2.31–17.14	8	9	7.0–12.1	3.07–14.97	4	13	6.4–10.5	1.91–7.50	27	3	5.5–9.0	1.56–6.76	4	11	5.7–11.0	1.80–12.68
September-17	7	7	6.6–11.4	2.62–13.91	5	13	5.8–10.8	2.12–12.67	4	10	5.3–9.9	1.33–7.31	3	28	5.8–10.6	1.92–10.06	7	12	7.1–10.5	3.36–10.65
October-17	7	8	6.5–11.5	2.18–12.47	9	9	5.7–10.5	1.61–10.19	6	14	6.6–9.6	2.53–8.08	5	14	6.1–9.8	2.2–6.93	4	22	6.4–10.4	2.09–10.57
November-17	12	12	6.4–10.7	2.08–10.26	9	8	5.5–11.2	1.43–10.49	10	6	6.7–11.2	1.80–9.15	9	13	5.6–9.5	1.46–7.49	13	13	6.3–10.5	2.00–9.15
December-17	7	16	6.7–11.3	2.66–11.92	13	15	5.7–11.4	1.31–12.38	10	19	7.2–11.3	3.18–13.73	16	10	5.5–8.5	1.50–5.76	16	15	6.0–11.0	1.77–9.48
January-18	10	14	6.7–11.5	2.45–13.15	2	8	6.6–11.1	2.16–11.32	12	14	6.0–10.9	2.64–11.87	10	7	6.1–9.7	1.95–8.68	11	11	5.8–8.8	1.85–5.43
February-18	5	9	7.2–11.2	2.96–12.48	9	12	6.8–9.6	2.51–6.35	10	19	7.2–11.3	3.18–13.73	6	15	6.1–9.0	1.63–6.85	7	13	6.0.3–9	2.53–6.11
March-18	19	20	6.9–11.6	2.76–15.06	3	9	6.9–11.3	2.77–11.52	3	11	7.2–11.5	3.25–14.2	10	25	5.4–9.6	1.21–8.25	10	12	5.0–11.2	1.14–15.00
April-18	11	24	7.2–11.3	3.10–12.40	7	15	5.5–11.5	1.47–13.03	20	12	5.0–10.1	1.13–9.19	12	24	5.4–9.5	1.39–7.21	12	18	6.7–10.0	2.2–8.28
May-18	6	6	6.0–11.6	1.73–13.04	11	17	6.5–10.5	2.13–11.05	3	30	6.2–11.2	2.01–12.53	9	20	6.4–11.6	2.32–13.75	2	30	6.0–10.8	1.74–10.57
June-18	6	13	6.6–10.9	2.31–12.25	13	20	7.0–11.2	3.11–13.90	10	16	5.5–10.5	1.58–9.92	17	14	6.8–10.6	2.91–11.33	7	28	7.7–10.5	3.29–10.7
July-18	2	28	7.0–11.3	2.69–12.24	13	18	7.0–11.2	2.77–12.35	12	17	6.9–10.5	2.83–10.34	12	18	5.5–10.7	1.46–11.79	11	19	6.0–10.5	2.04–9.4
August-18	15	15	6.3–11.5	2.20–17.14	15	15	5.5–12.1	1.46–14.97	6	24	6.7–11.0	2.42–11.17	27	3	5.5–9.0	1.56–6.76	5	25	5.8–11.6	2.01–12.86
September-18	7	23	7.0–10.3	2.76–11.46	8	22	7.1–11.4	2.96–11.93	6	24	6.3–11.4	2.36–13.66	7	23	5.6–10.8	1.39–10.66	7	22	5.8–9.0	1.37–6.61
October-18	8	22	7.0–11.0	3.21–11.55	14	16	6.6–11.1	2.49–9.88	14	16	6.7–10.9	2.47–10.55	6	24	5.8–9.6	1.43–8.27	10	20	6.0–10.5	1.56–8.42
November-18	7	22	7.1–10.6	3.48–11.17	7	22	7.7–11.5	3.93–14.01	9	20	6.9–10.8	2.42–11.09	16	13	6.0–9.5	1.65–8.51	10	20	5.8–11.2	1.58–11.71
December-18	6	23	6.5–10.5	1.93–10.11	5	25	7.5–11.2	3.15–13.94	15	15	6.2–10.5	1.78–8.89	5	25	6.6–10.2	2.11–9.40	8	20	6.4–11	2.12–10.74
January-19	16	18	6.5–10.3	1.95–8.49	18	22	5.9–11.4	1.83–11.43	16	18	6.3–11.3	2.90–13.86	17	21	6.0–9.3	2.03–6.59	17	20	6.4–9.9	1.79–9.20
February-19	18	21	7.2–11.3	3.03–12.49	17	20	6.3–11.5	2.00–13.16	19	20	6.1–11.1	1.85–13.72	16	19	5.2–11.2	1.25–14.62	15	18	5.6–9.8	1.33–8.74
March-19	17	20	7.7–11.4	3.41–14.30	16	23	5.7–11.3	1.61–13.09	18	20	5.2–9.7	1.24–8.62	19	23	6.8–11.0	2.24–10.99	16	19	5.6–9.6	1.6–8.64
April-19	16	19	6.3–11.4	1.90–12.13	17	21	5.7–11.3	1.60–14.03	20	22	5.8–11.2	1.73–12.65	17	19	6.0–10.7	1.5–11.12	18	21	5.7–11.5	1.55–12.08
May-19	19	22	7.0–11.4	2.50–13.63	17	22	6.3–12.1	1.98–15.11	18	20	7.0–11.2	2.66–11.18	15	18	6.3–10.3	1.76–8.64	15	23	5.8–11.9	1.62–14.57
June-19	18	21	7.6–11.0	3.00–12.08	16	19	6.9–11.7	2.73–14.57	19	21	6.6–11.4	2.59–13.79	16	21	6.0–10.8	1.73–9.96	14	19	6.3–11	1.92–12.49
Total	252	396	5.6–11.6	1.39–17.14	265	384	5.5–12.1	1.31–15.11	271	416	5.0–11.5	1.13–14.2	307	420	5.2–11.6	1.21–14.62	245	461	5–11.9	1.14–15.00

The TL and W varied by sampling site and increased from brackish water in LPST to upstream in LXAG (*n*_W_ = 3,417, *df*_W_ = 3, *F*_W_ = 126.14, *p*_W_ < 0.001, *Cl*_*95%* W_ = 4.77–5.15; *n*_TL_ = 3,417, *df*_TL_ = 3, *F*_TL_ = 137.52, *p*_TL_ < 0.001, *Cl*_*95%* TL_ = 8.02–8.22). Specifically, W increased from 4.96 ± 0.12 at LPST to 5.93 ± 0.12 at CRCT, 6.81 ± 0.12 at TNCT and 7.17 ± 0.12 g at LXAG. Similarly, TL increased from LPST to CRCT, TNCT, and LXAG with values of 8.1 ± 0.1, 8.5 ± 0.1, 9.0 ± 0.1, and 9.2 ± 0.1 cm, respectively.

### Length-weight relationship and growth pattern

**Figure 2:**
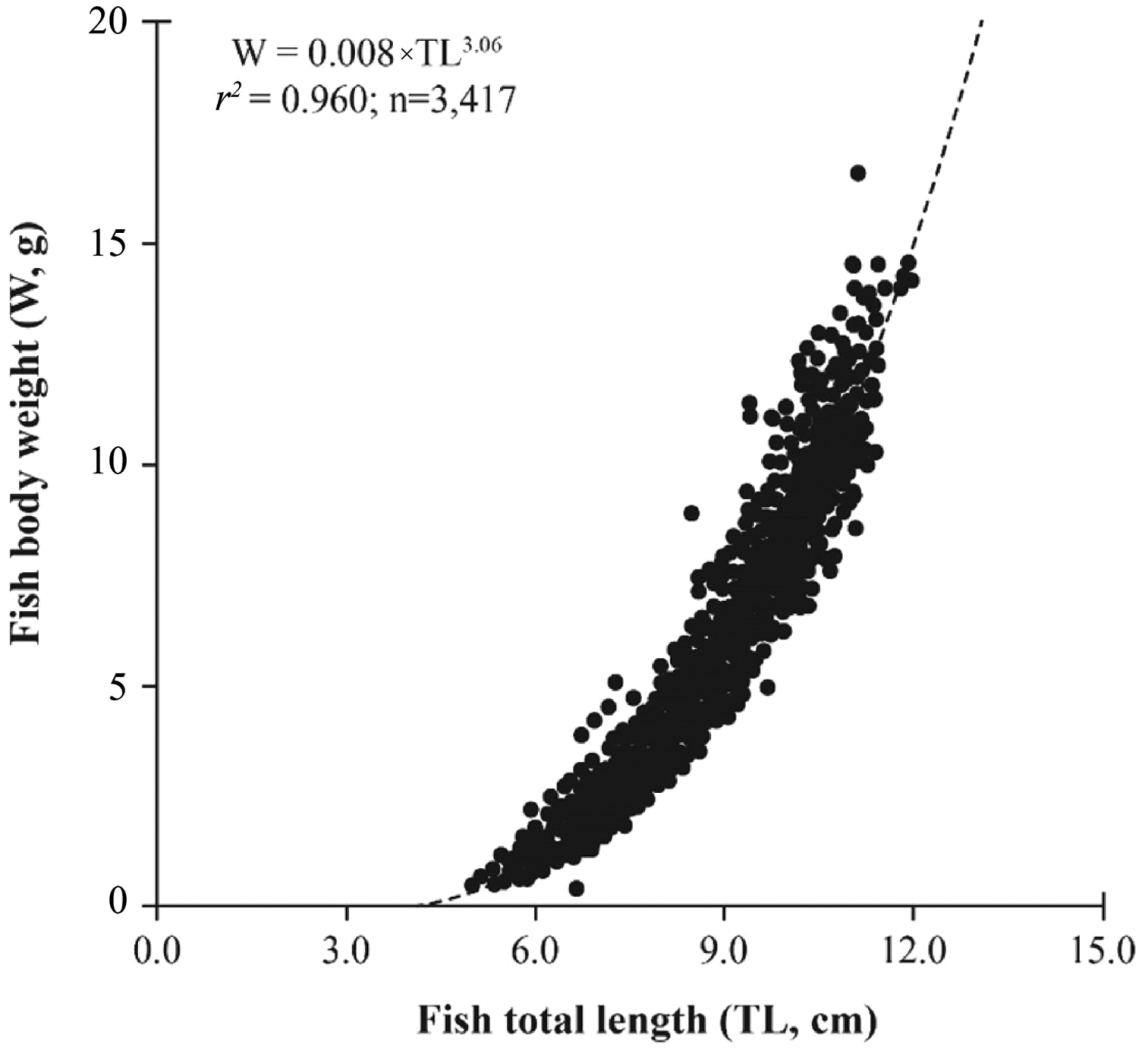
The relationship of total length to the weight in *Periophthalmdodon septemradiatus*.

**Figure 3:**
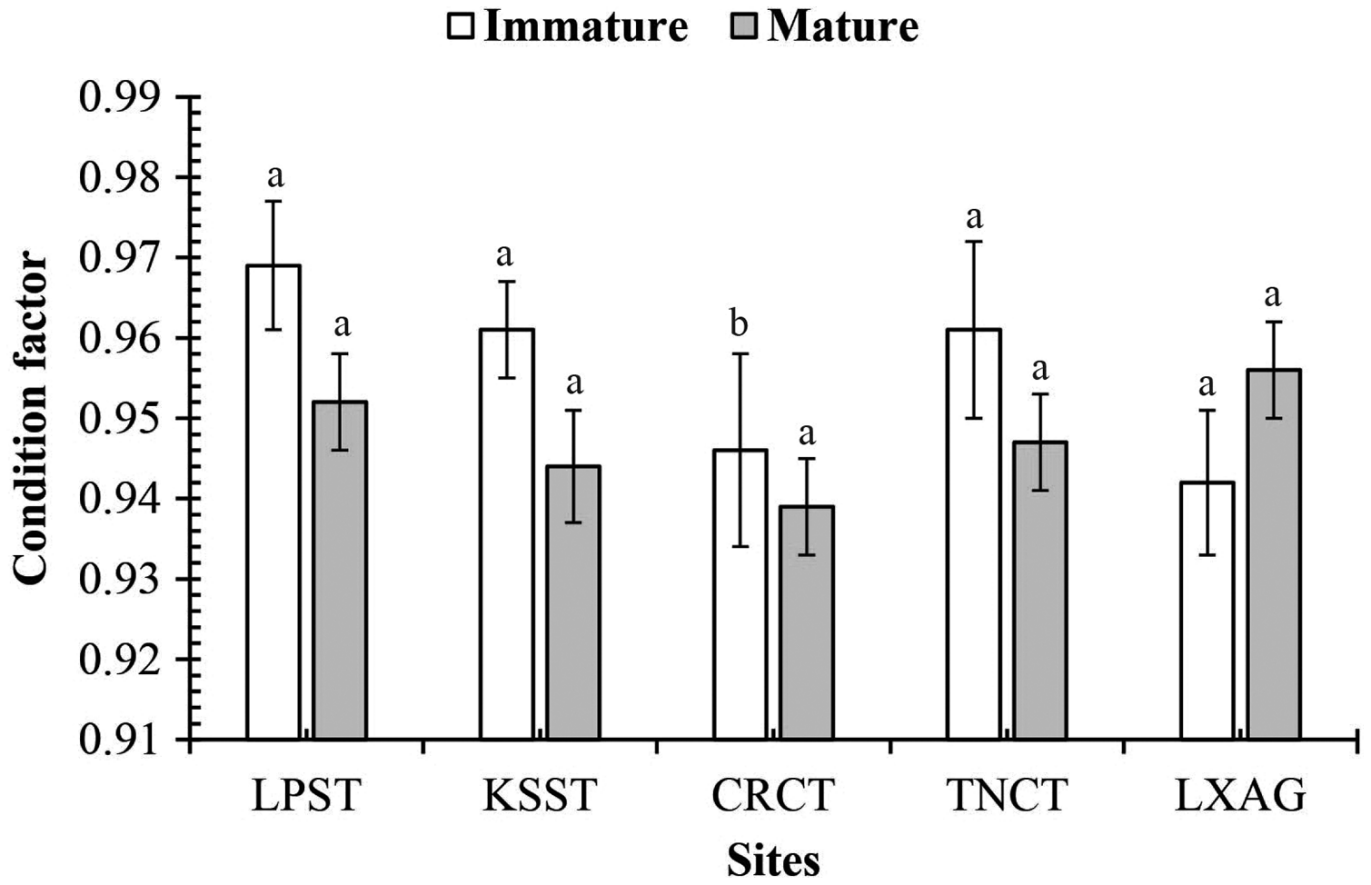
The condition factor of *Periophthalmodon septemradiatus* by fish size and site interaction. (LXAG, Long Xuyen–An Giang; TNCT, Thot Not–Can Tho; CRCT, Cai Rang–Can Tho; KSST, Ke Sach–Soc Trang; and LPST, Long Phu, Soc Trang; the vertical bar is the standard error of mean; a and b represent the significant difference).

The mudskipper showed a positive relationship between TL and W due high determination values (*r*^*2*^ = 0.92–0.98 in Table 2). The *b*-value of LWR was 3.06 ± 0.01 and larger than 3 (*n* = 3,417, *df* = 3145, *t* = 5.36, *p* < 0.001, Fig. 2) showed that this fish exhibited positive allometric growth type. The fish growth pattern varied by sex because males exhibitted positive allometry (*b* = 3.09 ± 0.01, >3; *n* = 2,077, *df* = 2,075, *t* = 6.14, *p* < 0.001) whereas females displayed isometry (*b* = 3.02 ± 0.02, = 3, *n* = 1,340, *df* = 1,338, *t* = 1.21, *p* = 0.23). The fish growth pattern also changed with fish size since the immature fish belonged to negative allometry (*b* = 2.91 ± 0.03, <3, *n* = 954, *df* = 952, *t* = −3.13, *p* < 0.001), while mature fish showed positive allometry (*b* = 3.09 ± 0.02, >3, *n* = 2,463, *df* = 2,461, *t* = 5.56, *p* < 0.001) (Fig. 3).

**Table 2:** Growth pattern of *Periophthalmodon septemradiatus* in 24 months (I, isometry; P, positive allometry; *n*, number of individuals; *b*, growth parameter; *a*, slope coefficient; SE, standard deviation).

Month	*n*	*r* ^2^	*b*	SE	*a*	SE	Growth type	*t*	*p*
July-17	116	0.96	3.17	0.06	0.006	0.001	P	2.71	0.01
August-17	94	0.96	2.97	0.06	0.009	0.001	I	−0.52	0.61
September-17	96	0.96	3.15	0.06	0.007	0.001	P	2.30	0.02
October-17	98	0.97	3.03	0.06	0.008	0.001	I	0.58	0.56
November-17	105	0.96	2.91	0.06	0.010	0.001	I	−1.50	0.14
December-17	137	0.97	2.95	0.04	0.009	0.001	I	−1.19	0.24
January-18	99	0.93	2.96	0.08	0.010	0.002	I	−0.49	0.62
Febuary-18	105	0.95	3.10	0.07	0.007	0.001	I	1.44	0.15
March-18	122	0.97	3.13	0.05	0.007	0.001	P	2.79	0.01
April-18	155	0.97	2.98	0.04	0.009	0.001	I	−0.56	0.58
May-18	134	0.98	3.17	0.04	0.006	0.001	P	3.86	0.00
June-18	144	0.96	2.96	0.05	0.010	0.001	I	−0.81	0.42
July-18	150	0.97	3.04	0.04	0.008	0.001	I	0.95	0.34
August-18	150	0.98	2.94	0.04	0.010	0.001	I	−1.56	0.12
September-18	149	0.96	3.28	0.06	0.005	0.001	P	4.83	0.00
October-18	150	0.95	3.07	0.06	0.007	0.001	I	1.25	0.21
November-18	146	0.97	3.06	0.04	0.007	0.001	I	1.47	0.15
December-18	147	0.96	3.13	0.05	0.006	0.001	P	2.44	0.02
January-19	183	0.92	2.99	0.07	0.008	0.001	I	−0.09	0.93
Febuary-19	183	0.96	3.17	0.05	0.006	0.001	P	3.72	0.00
March-19	191	0.96	3.07	0.05	0.007	0.001	I	1.44	0.15
April-19	190	0.97	3.12	0.04	0.007	0.001	P	2.93	0.00
May-19	189	0.97	3.15	0.04	0.006	0.001	P	3.54	0.00
June-19	184	0.94	2.98	0.06	0.009	0.001	I	−0.34	0.73
Total	3,417	0.96	3.06	0.01	0.008	0.000	P	5.36	0.00

The growth pattern of fish shifted from isometric growth type in the dry season because *b* (3.02 ± 0.02) was equal 3 (*n* = 646, *df* = 644, *t* = 0.96, *p* = 0.34) to positive allometric one in the wet season as *b* (3.07 ± 0.01) was >3 (*n* = 2,771, *df* = 2,769, *t* = 5.75, *p* < 0.001). This mudskipper displayed isometry at LPST (*b* = 3.03 ± 0.03, ≈3, *n* = 706, *df* = 704, *t* = 1.12, *p* = 0.27) and KSST (*b* = 2.99 ± 0.03, ≈3, *n* = 727, *df* = 725, *t* = −0.36, *p* = 0.72), but positive allometry at CRCT (*b* = 3.08 ± 0.03, >3, *n* = 687, *df* = 685, *t* = 2.78, *p* = 0.01), TNCT (*b* = 3.06 ± 0.03, >3, *n* = 649, *df* = 647, *t* = 2.52, *p* = 0.01) and LXAG (*b* = 3.16 ± 0.03, >3, *n* = 648, *df* = 646, *t* = 6.52, *p* < 0.001). In most months, the growth pattern of *P. septemradiatus* exhibited isometry with a growth parameter *b* < 3 (*p* < 0.001 for all cases). However, in September-17, March-18, May-18, September-18, December-18, February-19, April-19, and May-19 displayed positive allometry with *b* > 3 (Table 2).

### Condition factor (CF)

The condition factor of *P. septemradiatus* was 0.95 ± 0.00 and significantly less than 1 the well-being threshold (*n* = 3,417, *df* = 3,416, *t* = −28.73, *p* < 0.001, *CI*_*95%*_ = (−0.06) to (−0.05)). The CF did not vary by sex (*n* = 3,417, *df* = 3,415, *t* = 1.17, *p* = 0.24, *CI*_*95%*_ = 0.00–0.01), fish size (*n* = 3,417, *df* = 3,415, *t* = −0.79, *p* = 0.43, *CI*_*95%*_ = (−0.01) to 0.00) and season (*n* = 3,417, *df* = 3,415, *t* = 1.07, *p* = 0.29, *CI*_*95%*_ = (0.00)–0.01). The mudskipper CF changed with sampling site, showing lowest value of 0.94 ± 0.01 at LPST, KSST, TNCT and LXAG and highest value of 0.97 ± 0.01 at TDST (*n* = 3,417, *df* = 3, *F* = 6.26, *p* < 0.001, *CI*_*95%*_ = 0.94–0.95). Similar to the studied months, CF was less than 1 (*p* < 0.001 for all cases), except for September-17 months. (*n* = 96, *df* = 95, *t* = 1.01, *p* = 0.32, *CI*_*95%*_ = (−0.01) to 0.03), January-18 (*n* = 99, *df* = 98, *t* = −0.35, *p* = 0.73, *CI*_*95%*_ = (−0.03) to 0.02), and March-18 (*n* = 122, *df* = 121, *t* = −0.72, *p* = 0.47, *CI*_*95%*_ = (−0.02) to 0.01) (Table 3).

**Table 3:** Condition factor of *Periophthalmodon septemradiatus* collected from five sites.

Month	*n*	Mean	SE	*t*	*df*	*p*
July-17	116	0.91	0.01	−7.90	115	<0.001
August-17	94	0.97	0.01	−2.44	93	0.02
September-17	96	1.01	0.01	1.01	95	0.32
October-17	98	0.96	0.01	−5.22	97	<0.001
November-17	105	0.93	0.01	−7.95	104	<0.001
December-17	137	0.95	0.01	−7.21	136	<0.001
January-18	99	1.00	0.01	−0.35	98	0.73
Febuary-18	105	0.95	0.01	−4.89	104	<0.001
March-18	122	0.99	0.01	−0.72	121	0.47
April-18	155	0.97	0.01	−4.26	154	<0.001
May-18	134	0.94	0.01	−7.98	133	<0.001
June-18	144	0.98	0.01	−2.26	143	0.03
July-18	150	0.96	0.01	−6.10	149	<0.001
August-18	150	0.98	0.01	−2.14	149	0.03
September-18	149	0.95	0.01	−4.76	148	<0.001
October-18	150	0.91	0.01	−10.73	149	<0.001
November-18	146	0.94	0.01	−9.06	145	<0.001
December-18	147	0.91	0.01	−14.15	146	<0.001
January-19	183	0.92	0.01	−8.68	182	<0.001
Febuary-19	183	0.96	0.01	−4.73	182	<0.001
March-19	191	0.92	0.01	−10.88	190	<0.001
April-19	190	0.96	0.01	−5.69	189	<0.001
May-19	189	0.92	0.01	−13.61	188	<0.001
June-19	184	0.94	0.01	−8.11	183	<0.001
Total	3,417	0.95	0.01	−28.73	3,416	<0.001

**Figure 4:**
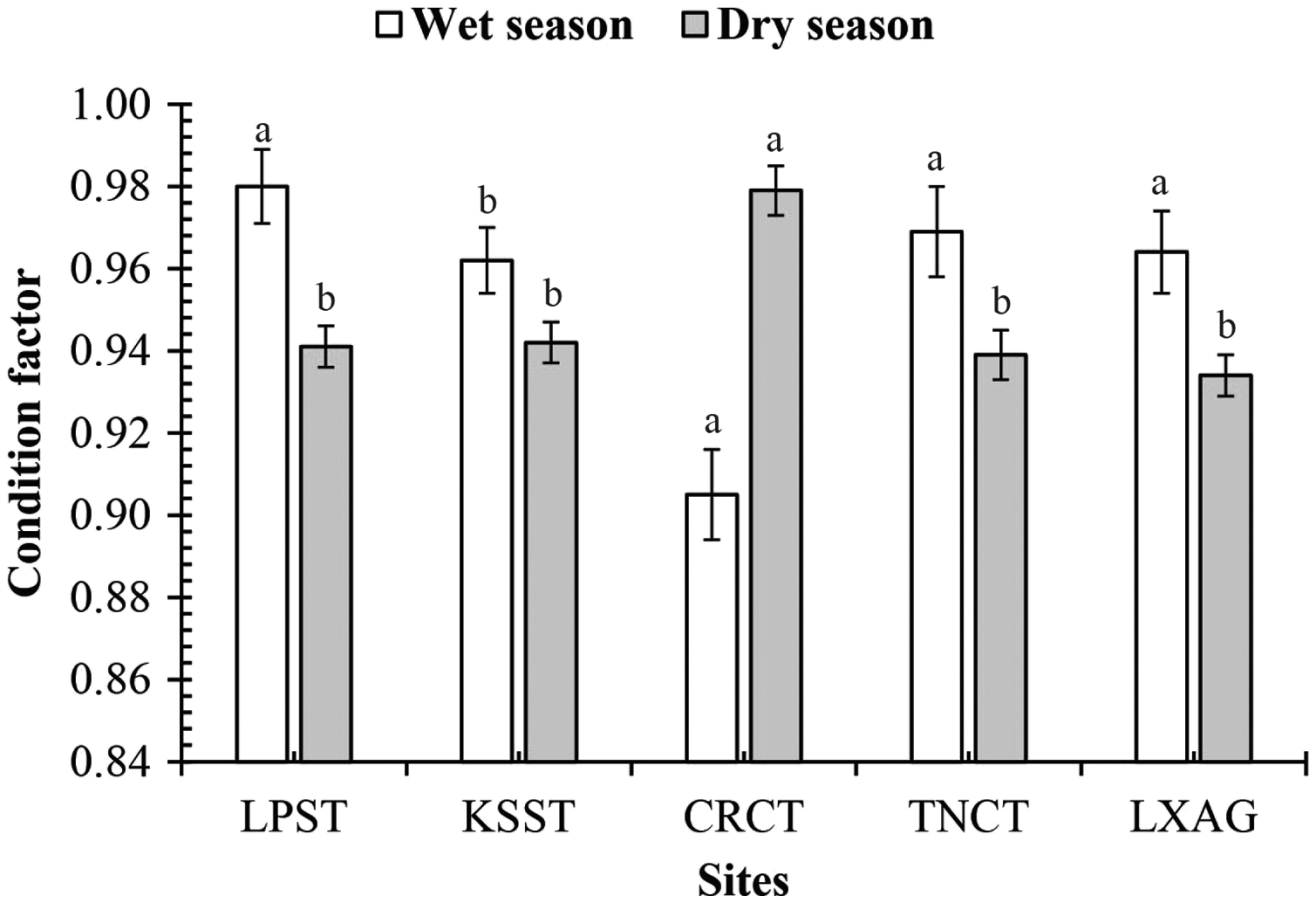
The condition factor of *Periophthalmodon septemradiatus* by season and site interaction. (LXAG, Long Xuyen–An Giang; TNCT, Thot Not–Can Tho; CRCT, Cai Rang–Can Tho; KSST, Ke Sach–Soc Trang; and LPST, Long Phu, Soc Trang; the vertical bar is the standard error of mean; a and b represent the significant difference).

The CF of this mudskipper was not regulated by interactions: sex × fish size (*df* = 1, *F* = 0.43, *p* = 0.51), sex × season (*df* = 1, *F* = 0.22, *p* = 0.64), sex × site (*df* = 4, *F* = 2.52, *p* = 0.04), fish size × season (*df* = 1, *F* = 0.21, *p* = 0.65), sex × fish size × season (*df* = 1, *F* = 0.90, *p* = 0.34), sex × fish size × site (*df* = 4, *F* = 1.79, *p* = 0.13), sex × season × site (*df* = 4, *F* = 1.72, *p* = 0.14), fish size × season × site (*df* = 4, *F* = 1.60, *p* = 0.17), and sex × fish size × site (*df* = 4, *F* = 0.59, *p* = 0.67). However, the CF is changed by fish size × site (*df* = 4, *F* = 5, *p* < 0.001, Fig. 3) and season × site (*df* = 4, *F* = 8.61, *p* < 0.001, Fig. 4).

## Discussion

The total length and weight were higher in males and differed significantly sex. It could be caused by the female eating rarely during the egg caring and releasing period because the eggs occupy most of the abdominal cavity, whereas the male needs more energy to play a role in digging and protecting the burrow, as Dinh et al. (2020a) noted. This can lead to a slower growth rate in females than males. The TL and W changed by sex in this species were similar to those in *P. chrysospilos* in VMD (Dinh et al., 2022d) and *B. boddarti* in India (Mahadevan & Ravi, 2018). However, *P. novemradiatus* in India showed opposite results since the TL and W of females were more significant than males.

The LWR estimates for the variables in Table 2 should be considered tentative because of the relatively low values of the coefficient of determination (<0.95). The findings should be considered significant in the current study for variables that exhibit a high level of correlation (*r*^*2*^). The mudskipper *P. septemradiatus* exhibited high correlation between TL and W (*r*^*2*^ = 0.92–0.98), indicating that W was estimated from a fish given length. Some other mudskippers living in and out of the VMD also demonstrated a high correlation in LWRs, *e.g*., *B. boddarti* in VMD and India (Dinh, 2017b; Mahadevan & Ravi, 2018), *P. novemradiatus* in India (Mahadevan & Ravi, 2018), *P. variabilis* in VMD (Dinh et al., 2022b) and *P. gracilis* in VMD (Dinh et al., 2022c). The high correlation in LWRs was also found in some other gobies in the VMD, *e.g*., *Glossogobius sparsipapillus* (Dinh, 2015; Truong et al., 2021), *Butis butis* (Dinh, 2017a) and *G. giuris* (Dinh & Ly, 2014; Phan et al., 2021b).

Since *b* was >3, *P. septemradiatus* displayed a dominant growth in fish high (*e.g*., fish depth) over TL as fish grew, and almost all specimens were collected at the mature stage. The mudskipper growth pattern varied by sex due to the difference in the gonadal weight of males and females. The sexual change in growth pattern was found in *P. modestus* in northern Viet Nam (Tran, Nguyen & Ha, 2021b), but not *P. novemradiatus* in India (Mahadevan & Ravi, 2018), *B. boddarti* in VMD and India (Dinh, 2017b; Mahadevan & Ravi, 2018) and *P. gracilis* in VMD (Dinh et al., 2022c). The species *P. septemradiatus* displayed intraspecific change since its growth type shifted from negative allometry in the immature fish to positive allometry in mature fish, suggesting that the energy needs increased as fish grow. This assumption could be caused by the variation in food feeding and composition between these two fish groups, as Dinh et al. (2020b) reported. Likewise, the growth pattern *P. gracilis* in VMD was also regulated by fish size (Dinh et al., 2022c). Males exhibited positive allometry (*b* > 3), whereas females displayed an isometric pattern (*b* = 3). The difference in food composition of *P. septemradiatus* between the wet and dry seasons documented by Dinh et al. (2020b) could influence the fish growth pattern. The mudskipper showed isometry in the dry season when fish consumed four types of food item types and positive allometry in the wet season when fish ingested six types of food. Likely, *P. modestus* in northern Viet Nam (Tran, Nguyen & Ha, 2021b) and *P. gracilis* in VMD (Dinh et al., 2022c) exhibited the seasonal change in growth pattern. The variation in abiotic (*e.g*., salinity and mudbank slope) and biotic (*e.g*., vegetation) factors between these five sampling sites (Dinh et al., 2021) could lead to the spatial change in *P. septemradiatus* growth pattern. This assumption was also found in *P. modestus* in northern Viet Nam (Tran, Nguyen & Ha, 2021b) and *P. gracilis* in VMD (Dinh et al., 2022c). The spatial change in growth pattern was not found in some other species of Oxudercidae like *P. schlosseri* (Dinh, 2016) and *P. chrysospilos* (Dinh et al., 2022d). The species *P. septemradiatus* displayed a temporal change in growth type, shifting from isometry to positive allometry. This assumption was also found in *P. schlosseri* (Dinh, 2016), *P. chrysospilos* (Dinh et al., 2022d), *P. variabilis* (Dinh et al., 2022b), and *P. gracilis* (Dinh et al., 2022c).

The growth pattern generally of *P. septemradiatus* was inconsistent with the results in Malaysia, where it exhibited negative allometry as (*b* = 2.90) was <3 documented by Khaironizam & Norma-Rashid (2002). The difference in fish growth patterns between the present and previous studies could be regulated by the difference in environmental factors, size range and sampling size (22 individuals in the previous research and 3,417 individuals in the present study). The positive allometry was also found in some other mudskippers like *P. chrysospilos* in VMD (*b* = 3.16) (Dinh et al., 2022d) and Malaysia (*b* = 3.21) (Che Abdullah & Md Zain, 2019), *P. modestus* in northern Viet Nam (*b* = 3.10) (Tran, Nguyen & Ha, 2021b), *P. argentilineatus* (*b* = 3.34), *P. spilotus* (*b* = 3.50), and *P. schlosseri* (*b* = 3.06) in Malaysia (Khaironizam & Norma-Rashid, 2002). By contrast, *P. schlosseri*a congener of *P. septemradiatus* living in the mudflats on the coastline of VND showed an isometry (*b* = 2.96) (Dinh, 2016). Besides, some other mudskippers distributed mudflats in the estuarine and coastal regions like *P. variabilis* in VMD (Dinh et al., 2022b), *P. waltoni* in Sourgalm, Iran (*b* = 3.00) (Ghanbarifardi et al., 2014), *B. boddarti* (*b* = 3.00) (Khaironizam & Norma-Rashid, 2002) also displayed isometry. Some other mudskipper exhibited negative allometry, *e.g*., *P. gracilis* in VMD (*b* = 2.69) (Dinh et al., 2022c), *P. waltoni* in Mahshahr, Iran (*b* = 2.82) (Ghanbarifardi et al., 2014), *P. novemradiatus* (*b* = 2.67), *S. histophorus* (*b* = 2.62) in Malaysia (Khaironizam & Norma-Rashid, 2002). The *b* value of LWR was generally expected to be in the range of 2.5–3.5 for fish (Carlander, 1977), and *b* values can be affected by environmental conditions and sampling location *b* value within the expected range (with a mean of 3.06) at all sampling times in this study. This result indicates a good match between TL and W. Even in some previous studies, this harmony is not seen (*S. tenuis* (*b* = 2.47) and *B. dussumieri* (*b* = 2.37) in Qeshm, Iran; Ghanbarifardi et al., 2014). It consequently showed that the growth pattern depends on each different environment and each specific species.

According to Dinh et al. (2020b), this species feeds on mudflats along the river. However, the area of natural alluvial grounds is shrinking due to sea-level rise, and human activities are also narrowing the mudflats in VMD. These affect the habitat of this fish. With CF = 0.95, this fish species has not yet adapted to the change of habitat. The fish developmental stages could affect the CF of *P. gracilis* (Dinh et al., 2022c) but not the CF of *P. septemradiatus* because this value did not vary by sex and size. In contrast, the sexual variation in CF was in *P. serperaster* in VMD (Dinh et al., 2016). The species *G. giuris* (Phan et al., 2021b) in VMD did not exhibit an intraspecific change. The seasonal change did not regulate the seasonal change in CF of *P. septemradiatus* since the fish shared the same pattern in CF in the dry-wet season pattern. This assumption was also found *P. elongatus* (Tran, 2008). By contrast, *P. gracilis* in VMD lived well to the wet season (Dinh et al., 2022c). Like growth patterns, the variation in biotic and biotic factors among these five sampling sites could regulate CF variations of *P. septemradiatus*. The CF’s spatial variation was likely found in *P. gracilis* in VMD (Dinh et al., 2022c).

## Conclusion

The findings provided basic information on the growth and adaptation of *P. septemradiatus* being helpful in fish adaptation understanding and resource conservation in VMD. The environment at VMD has undergone drastic changes in recent years and is strongly affecting the growth and adaptation of this species. This information is essential for the future cultivation of this species and suggests a reasonable strategy to conserve them.

## Supplemental Information

10.7717/peerj.13880/supp-1 Supplemental Information 1 Raw *Periophthalmodon septemradiatus* data.

## Additional Information and Declarations

### Field Study Permissions

The following information was supplied relating to field study approvals (*i.e*., approving body and any reference numbers): In terms of the field trip, our research has no field permit requirement as we collect fish from the public-owned where local fishers collect fish daily. Moreover, we collect fish just once a month at each site, and the number of fish collection is within allowable limits of the animal assessment.

### Data Availability

The following information was supplied regarding data availability: Raw data is available in the Supplemental Files.
